# Electrons and Phonons in Pentacene: Coupling Patterns Reveal the Microscopic Origin of the Phonon Limited Mobility

**DOI:** 10.1021/acs.jpcc.5c04906

**Published:** 2025-11-29

**Authors:** Luca Gnoli, Elisabetta Venuti, Tommaso Salzillo, Matteo Masino, Patrizio Graziosi

**Affiliations:** † 9327 ISMN—CNR, Consiglio Nazionale Delle Ricerche, via Gobetti 101, 40129 Bologna, Italy; ‡ Dipartimento di Chimica Industriale “Toso Montanari”, Università di Bologna, via Gobetti 85, 40129 Bologna, Italy; § Dipartimento di Scienze Chimiche, Della Vita e Della Sostenibilità Ambientale & INSTM-UdR Parma, Parco Area Delle Scienze, 17/A, 43124 Parma, Italy

## Abstract

We present a comprehensive computational study of the vibrational properties and electron–phonon couplings in the three known polymorphs of pentacene. Vibrational patterns and electron–phonon interactions were evaluated at several ***q***-points of the Brillouin zone, allowing for the detailed mapping of the phonon landscape and the coupling mechanisms relevant to charge transport. Using a pool of postprocessing tools, we analyze the different phonon dispersions and show how low-frequency phonons modulate transport differently in each polymorph through distinct electron–phonon coupling (EPC) signatures in the reciprocal space. In particular, we demonstrate that different phonons dominate in the high-temperature and thin-film polymorphs compared with the high-mobility low-temperature polymorph, leading to different decoherence and localization trends. We describe the microscopic origin of mobility in the bulk phase, showing that polymorphism affects not only the transfer integrals but also the phonon spectrum. Importantly, we find that mobility is not limited by a single “killer” mode but rather by multiple phonons with diverse wave-vectors. We further explain how phonon confinement accounts for the enhanced mobility of the 2D phase. Finally, we address the frequent coexistence of multiple polymorphs in organic crystals, considering the implications of intergrowth, structural defects, and disorder.

## Graphical abstract

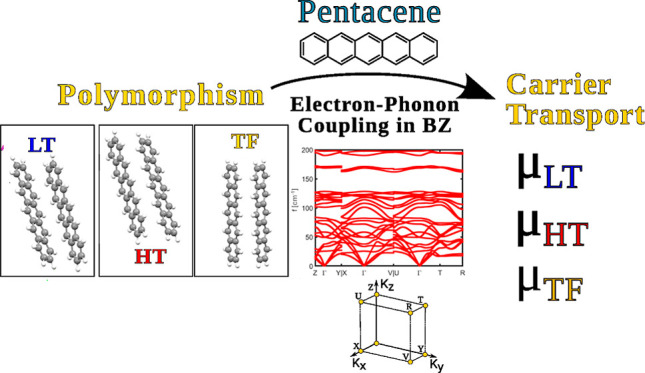

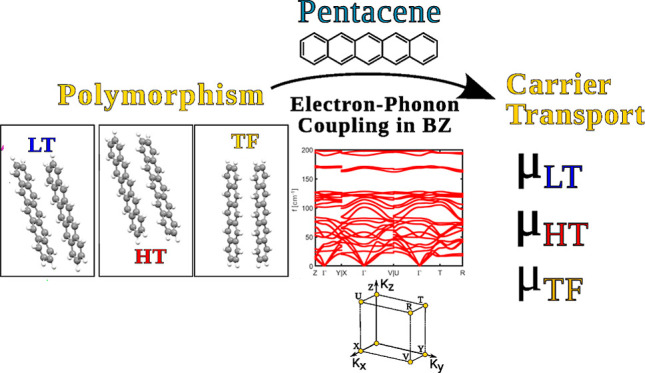

## 1 Introduction

Pentacene has been regarded for a long time as a benchmark for organic semiconductors (OSCs). Although in recent years research on OSCs has advanced toward novel functionalized systems, pentacene remains an ideal reference system for developing fundamental knowledge, as it is considered a model p-type semiconductor.^1–4^ This is of special importance because the widespread application of OSCs in electronics-related applications is hampered by the lack of predictive understanding of the inter-relationship between solid-state packing and device performance, irrespective of the specific type of the device. This challenge also arises from the likely occurrence of polymorphism, where a single molecular compound can crystallize in multiple distinct structures, each potentially exhibiting different electronic and vibrational properties. Thus, each polymorph can exhibit distinct variations in the electron–phonon coupling (EPC), thereby influencing the transport characteristics of the OSC.^5,6^ Indeed, low-frequency vibrational modes, such as translations and rotations of the entire molecule, play a pivotal role in introducing the dynamic disorder, which, in turn, affects the electronic transfer integrals and hence the bandwidth, ultimately shaping the macroscale electronic properties of the semiconductor. Therefore, understanding polymorphism is crucial for defining the electronic properties of OSCs at the macroscopic scale.

With pentacene being a prototype organic semiconductor (OSC), many works have addressed the theoretical study of its charge mobility and the dynamic disorder caused by electron–phonon coupling (EPC). On the experimental side, considerable attention has been devoted to the effect of polymorphism on charge transport properties. However, relatively few studies have theoretically examined the differences between the various crystal phases.

Girlando et al.^7^ used a dimer model to link transfer integrals to lattice vibrations, showing that mobility in pentacene is limited by low-frequency phonons via a dynamic disorder in both low-temperature and high-temperature polymorphs. Semiempirical dimer calculations combined with lattice-dynamics simulations, validated against low-frequency Raman spectra, revealed polymorph-dependent EPC strengths. Landi et al.^1^ investigated how polymorphism influences charge transport by combining molecular dynamics with electronic structure calculations to evaluate transfer integral fluctuations, showing that differences in crystal packing strongly affect the dynamic disorder and that nonlocal EPC exhibits a clear temperature dependence. Both works were restricted to Γ-point modes.

In 2012, Yi et al.^8^ extended EPC analysis beyond the Γ-point, investigating phonon effects throughout the Brillouin zone. Using molecular mechanics supercell calculations, they provided a comprehensive picture of EPC in the pentacene low temperature (LT) polymorph, which remains the most common bulk phase. Perroni et al.^9^ modeled spectral and transport properties of oligoacenes using the band theory with explicit EPC couplings, showing that both inter- and intramolecular vibrations are essential to describe charge transport. Chang and Bernardi^10^ used the Boltzmann transport equation with first-principles EPC interactions to describe band-like transport in the weak-coupling regime, accounting for all phonon modes and electronic bands in organic molecular crystals including LT pentacene. Ciuchi et al.^11^ achieved excellent agreement with experimental photoemission data by incorporating molecular vibrational modes and disorder in their calculations. Together, these studies highlight the importance of comprehensive EPC models in explaining the complex charge transport behavior of organic semiconductors like pentacene.

In this study, we first present accurate and validated first-principles calculations of the three known pentacene polymorphs: low temperature (LT), high temperature (HT), and thin film (TF). We use the inherent structure method^12–15^ to ensure they are really three different structures and assess their vibrational fingerprints. Then, we compute the EPC at the zone-boundary and demonstrate that the strongest EPC happens for modes having nonzero momentum, i.e., not in Γ. We can then assign the experimentally identified modes, a task still missing so far. Finally, we use the computed EPC to evaluate the mobility μ^16^ of the three polymorphs and related mixture.^17^ Also, we used pentacene as a benchmark to test the computational description of the role of disorder or defects in terms of energy potential and carrier scattering.

## 2 Computational Method

### 2.1 Electronic and Vibrational Properties

We employ PBE–PAW pseudopotentials and the D3-BJ Grimme with Becke-Johnson damping functions for a posteriori VdW correction *at any stage* of a DFT calculation with VASP.^16,18–20^ We start from the experimental unit cell parameters and determine the optimal ***k***-point grid using a cutoff energy of 400 eV; ***k***-point samplings of 6 × 4 × 2, 8 × 6 × 3, and 8 × 6 × 3 proved to be adequate to achieve energy convergency, for the HT, LT, and TF polymorphs, respectively. After identifying a converging ***k*** *-*grid, we optimize the energy cutoff for the wave functions increasing the energy up to 1100 eV. Energy cutoff values of 900, 800, and 800 eV for the HT, LT, and TF polymorphs, respectively, in combination with the optimal *k*-grids above, proved to be adequate with a convergence around 0.1 meV/atom. Finally, we relax the atomic positions, keeping constant the unit cell parameters at their experimental values with target convergence criteria of 10^–8^ eV on the energy and 10^–6^ eV/Å on the forces. For the inherent structure study, we relax also the lattice parameters. Following relaxation, the Phonopy package^21,22^ is used to compute and diagonalize the dynamical matrix using a 2 × 2 × 2 supercell—a setup validated to ensure the convergence in the phonon frequency and 3D density of states (DOS) with no negative frequency, except for the acoustic branches in Γ, where a small negative frequency around 10^–1^ to 10^–2^ THz may appear and be considered acceptable. Finally, the off-resonant Raman activities are computed with the vasp_Raman.py program.^23^ This program uses VASP as back-end to compute the polarizability with the finite displacement approach and returns the Raman activity of the selected modes.

Throughout the text, we use ***k*** and ***q*** to indicate the BZ points and hence their wave-vector of electrons and phonons, respectively.

### 2.2 Electron–Phonon Coupling

We adopt a recently developed protocol to parametrize the EPC.^16^ The unit cell is modulated along the selected eigenmodes at the Γ point and all the relevant high-symmetry ***q*** **-**points within the Brillouin zone (BZ), as defined in the Bilbao Crystallographic Data Center.^24,25^ Therefore, a unit cell distorted along the eigenmode vibrational coordinate is generated for each considered ***q*** point and ν phonon branch. The Phonopy code automatically accounts for the atomic mass weights in this process. Considering that in the tight-binding approximation, the electronic energy dispersion along a 1D direction can be approximated to 2*t*_W_ cos­(*k*·*a*) that leads to a bandwidth *B*_W_ related to the transfer integral *t*_W_ by the relation *B*_W_ = 4*t*_W_.^26^ As a result, the comprehensive bandwidth across the full 3D BZ, and hence the corresponding transfer integral can be obtained from a first-principles DFT electronic structure calculation.

Under this conceptual framework, and taking into account both the traditional approach to EPC^27,28^ and the conventional definition of deformation potentials,^29,30^ the EPC constant for each ***q**-*point and phonon branch ν can be defined as^16^

1 $$D_{q,v}^{n,n}=\frac{\partial t_{W}^{n}}{\partial r_{q,v}}$$

2 $$D_{q,v}^{n,m}=\frac{\partial \Delta_{s}^{n,m}}{\partial r_{q,v}}$$

In eqs 1 and 2, *n* and *m* are the band indexes, *t*_W_^*n*^ is the bandwidth of the band of index *n*, Δ_s_^*n*,*m*^ is the Davydov splitting between the bands *n* and *m*, computed as the energy difference between the energy barycenter of the bands, and *r*_*q*,*v*_ is the average displacements of all the atoms in the structure distorted along the eigenmode (*q*,*v*). Thus, eq 1 is related to intraband processes, i.e., it will be used when the carrier scattering involves initial and final states in the same band, while eq 2 is related to interband processes.

Operatively, after a self-consistent DFT calculation for each (***q***,ν) modulated/eigen-distorted structure, we perform a non-self-consistent calculation and save the obtained electronic structure in the bxsf format^31^ with the c2x code.^32^ Ad hoc routines were developed to extract the EPC parameters as in eqs 1 and 2. Finally, since we consider states and transitions in the whole 3D electronic structure, a DOS-weighted average of the computed EPC across the ***q***-points for each branch *v* was performed to extract band-index-specific EPC parameters to be used throughout the whole BZ

3 $$D_{n,m,\nu }=\frac{\sum_{q}D_{q,v}^{n,m}{DOS}_{q,\nu }}{\sum_{q}{DOS}_{q,\nu }}$$

The selection of the vibrational modes to be considered in the protocol relies on the phonon DOS specifications and is made by taking the modes, which are bundled in the DOS, before the first drop in the DOS. For pentacene, this encompasses the 20 modes of the lowest frequency, with a drop between 130 and 140 cm^–1^ according to the polymorph. The comprehensive EPCs parametrized in eq 3 are regarded as the proper deformation potential for inelastic processes involving the so-called nonpolar optical phonon in the mobility calculation, as detailed in the next subsection.

### 2.3 Mobility

#### 2.3.1 Proposed Methodology

Crystalline OSCs exhibit a well-defined crystal structure, which results in a distinctive vibrational fingerprint. Adopting a description based on the electronic dispersions in the BZ of the reciprocal lattice, the mobility μ is evaluated from the conductivity σ as

$$\mu_{ij\left(E_{F},T\right)}=\frac{\sigma_{ij\left(E_{F},T\right)}}{{n\cdot q}_{0}}$$ 4

where *i* and *j* are the Cartesian components *x*, *y*, and *z*, of the mobility and conductivity tensors, *E*_F_ is the Fermi level, *T* is the temperature, n is the carrier density, and *q*_0_ is the electronic charge.

The conductivity is computed in the context of the linearized BTE.^33^ In this context, the steady-state Boltzmann Transport Equation (BTE) is defined as^34^

5 $${\frac{\partial f}{\partial t}|}_{diff}+{\frac{\partial f}{\partial t}|}_{field}+{\frac{\partial f}{\partial t}|}_{coll}=0$$

where *f* is the distribution function of the system and the three terms are its time variation as due to diffusion, external field, and collisions, i.e., scattering events. Being *f* dependent on the position and electron momentum, the BTE tells that, at any point and for any wave vector, the total net rate of change in the electron distribution is zero. In the absence of thermal gradient and magnetic field, and under homogeneous electric field conditions, if we assume that the distribution function varies negligibly compared to the equilibrium, *f* = *f*_0_ + *g*, and keep only the terms linear in the electric field *E⃗*, the BTE becomes^34^

6 $$-\frac{\partial f_{0}}{\partial E}vfq_{0}\left|\mathrm{E⃗}\right|=-{\frac{\partial f}{\partial t}|}_{coll}$$

where *v* and *f* are the velocity and distribution functions of each charge carrier. We go further making the phenomenological assumption^34^

7 $$-{\frac{\partial f}{\partial t}|}_{coll}={-\frac{\partial \left(f_{0}+g\right)}{\partial t}|}_{coll}=\frac{1}{\tau }g$$

This approximation tells that, if we turn off the field, any out of balance term of the distribution decays to zero with a characteristic time τ; we have introduced a relaxation time τ, leading to the relaxation time approximation (RTA). In the context of periodic solids, where we define direct and reciprocal lattices, *v*, *f*, *g*, and τ depend on the specific electronic states of wave-vector ***k***.

Thus, under this approximation, the point becomes the computation of τ_(*k*)_. This derivation is quite cumbersome^34–37^ and goes way beyond the purpose of the present work. We just observe that the description in eqs 5–7 is quite general, and as long as the electron wave-vector ***k*** can be defined, this approach is formally applicable; the suitability of this picture is promoted by the accuracy in the reproduction of the experimental Raman spectra,^16^ which relies on a reciprocal lattice description.

Under the RTA, the conductivity is computed as^33^

8 $$\sigma_{ij\left(E_{F},T\right)}=q_{0}^{2}\int_{E}\Xi_{ij}\left(E\right)\left(-\frac{\partial f_{0}}{\partial E}\right)\;dE$$

The integrand of eq 8 contains the transport distribution function (TDF) Ξ_*ij*_ and the energy derivative of the equilibrium Fermi–Dirac distribution *f*_0_. The TDF is defined as

9 $$\Xi_{ij}\left(E\right)=\frac{2}{{\left(2\pi \right)}^{3}}\sum_{n}\sum_{k_{n,E}}v_{i,k_{n,E}}v_{j,k_{n,E}}\tau_{i,k_{n,E}}g_{k_{n,E}}$$

In eq 9, *v* is the band velocity, τ is the relaxation time, and *g* is the electronic DOS. All these quantities are specific to each individual transport state *k*_*n*,*E*_, where *k* is the wave-vector, *n* is the band index, and *E* its energy. So, the sum runs over all of the transport states identified by their wave-vector ***k***, belonging to all of the bands, having a certain energy. The DOS *g*_*k*_*n*,*E*__ is defined as $\frac{{dA}_{k_{n,E}}}{{\mathrm{v⃗}}_{k_{n,E}}\vee }$ , where d*A*_*k*_*n*,*E*__ corresponds to the area of the surface element of the constant energy surface to which the *k*_*n*,*E*_ state belongs, associated with each specific *k*_*n*,*E*_ state. Therefore, the sum in eq 6 is performed for each constant energy surface to compute the energy-dependent TDF, which will be then integrated as in eq 8. In this work, the tetrahedron method has been employed to construct the constant energy surfaces and extract the related quantities.^38,39^

The relaxation time of the state *k*_*n*,*E*_ for the transport along the direction *i*, associated with the scattering by a phonon belonging to the branch ν, is assessed from the inelastic scattering with nonpolar optical phonons as (we omit the index ν for clarity)^29,30,35,36,39^

10 $$\frac{1}{\tau_{i,k_{n,E}}}=\frac{1}{{\left(2\pi \right)}^{3}}\sum_{k_{n^{\prime },E^{\prime }}^{\prime }}\frac{\pi D_{n,n^{\prime }}^{2}}{\rho \omega_{0}}{\left(N_{\omega_{0}}+\frac{1}{2}\mp \frac{1}{2}\right)g}_{k_{n^{\prime },E^{\prime }}^{\prime }}\left(1-\frac{v_{i,k_{n^{\prime },E^{\prime }}^{\prime }}}{v_{i,k_{n,E}}}\right)$$

where $D_{n,n^{\prime }}$ is the deformation potential related to the electron–phonon scattering between the initial band *n* and the final band *n*′, which includes intra- and interband processes, evaluated from eqs 1 or 2, respectively; ρ is the mass density. The effective frequency ω_0,ν_ for the branch ν is evaluated from a DOS-weighted average over the selected portion of the phonon spectrum as $\omega_{0,\nu }=\frac{\sum_{\omega_{\nu }}\omega_{\nu }\cdot DOS\left(\omega_{\nu }\right)}{\sum_{\omega_{\nu }}DOS\left(\omega_{\nu }\right)}$ , where *N*_ω_0__ represents the phonon Bose–Einstein statistical distribution, while $g_{k_{n^{\prime },E^{\prime }}^{\prime }}$ is the DOS of the final state, belonging to the band *n*′ and at energy *E*′, which is either increased or decreased by ℏω for absorption or emission processes, respectively, denoted by “–” and “+” signs. The term $\left(1-\frac{v_{i,k_{n^{\prime },E^{\prime }}^{\prime }}}{v_{i,k_{n,E}}}\right)$ approximates the momentum relaxation time,^35,36,40^ which is the relevant type of relaxation time for computing transport coefficients.^30^ Note that this definition of relaxation time is similar to what appears in other works on OSCs.^41^ The calculation of the mobility is performed using the *ElecTra* simulator.^33,42^

Because the mobility tensor derived using equations is expressed in Cartesian coordinates, we project the electric field *E⃗* along the crystallographic axes. This is achieved by inverting the lattice vector matrix from the POSCAR VASP file

11 $$\vec{E_{l}}=l\times \mathrm{inv}\left(A\right)$$

where *l* is the intended direction in the internal coordinates and *A* is the lattice vector matrix. This allows us to express the electric fields along the internal cell axes, $\vec{E_{a}}$ , $\vec{E_{b}}$ , and $\vec{E_{c}}$ in Cartesian coordinates. Next, from the conductivity tensor σ́ , given by eq 8, we compute current density as

12 $$\vec{J_{l}}=\mathrm{\sigma ́}\vec{E_{l}}$$

Thus, we obtain a component of the conductivity tensor in internal coordinates

13 $$\sigma_{l}=\frac{\vec{J_{l}}}{\left|\vec{E_{l}}\right|}$$

and the related mobility

$$\mu_{l}=\frac{\sigma_{l}}{{n\cdot q}_{0}}$$ 14

Using this approach, we can link the computed mobility tensor in Cartesian coordinates to the mobility measured along specific crystal directions.

To perform the charge transport calculations, an additional nonself-consistent calculation on a finer mesh is needed. In this study, we adopted ***k*** *-*samplings of 28 × 21 × 11, 24 × 18, and 28 × 21 × 11, with cutoff energies of 800, 900, and 800 eV, for the LT, HT, and TF polymorphs, respectively. Importantly, the energy resolution used to construct the constant energy surfaces is set at 1 meV, meaning that each surface is calculated every 1 meV for each band, from the band edge up to ∼0.4 eV. Such a fine resolution proved to be essential for the treatment of the flatter bands of the OSC compared to inorganic compounds.^39,43–45^ Due to the large band gap of pentacene, we performed unipolar calculations separately for electron mobility in the conduction band (CB) and hole mobility in the valence band (VB).

#### 2.3.2 Discussion on the Methodology

By looking at the playground of charge transport models used in crystalline organic systems, we observe that sophisticated or computationally intensive approaches exist (e.g., mean field models, open quantum systems, quantum Monte Carlo, Transient Localization Theory—TLT), but in most cases, approximations leading to analytical expressions are used for practical purposes.^46^ These generally fall into two categories: hopping and band-like transport via the BTE. Moreover, the BTE approach, suitable for crystalline OSCs, is recognized to be precise when the electronic structure details are accounted for, but effective mass approximation is generally used to gain simpler equations.^47^ A conceptual bridge between these two macro categories is represented by TLT. This model considers both the effect of the periodic structure of the crystal as well as a possible carrier localization due to the dynamic disorder causing the vibrational modes.^11,48,49^

We observe that the thermal motion, responsible for the dynamic disorder used in TLT,^49^ is due to vibrational modes, especially the most populated intermolecular lattice phonons. The effect of the phonons in the modulation of the electronic properties is captured by eqs 1–3, and their population is considered in the relaxation time evaluation in eq 10. Thus, the approach we use considers the vibrational modes responsible for the thermal disorder, their population with temperature, and their effect on the electronic properties. What is missing in our method are the thermally induced band tails and the band narrowing responsible for the break of bands picture. Although we plan to include this aspect in the further development of our method, we reckon that as long as the electronic bands are present, our approach appears suitable. This is ensured by the proper computation of the Raman spectra, which could not reproduce the experimental spectra if the starting electronic structure was not reliable, and it is based on the electronic dispersions in the reciprocal lattice. Indeed, it is recognized that the band transport picture can catch relevant transport features in OSCs with intrinsic mobility above ∼0.5 cm^2^/(V s).^50^

Of relevance, in the TLT, is the relaxation time τ, which has also been introduced in the most recent implementations and generalizations of the hopping theories.^51^ Such τ is often considered to be constant, i.e., the independent of the energy/***k***-vector of the charge carrier (hole or electron), for example, it has been used ℏ/τ = ℏ/ω_0_ = 5 meV regardless of the molecular system.^51,52^ Thus, in the conceptual framework of these models, the precise determination of the charge diffusion length may suffer from an uncertainty in the characteristic times.

We relax these approximations by adopting a simulation approach capable of an atomistic description of the charge transport in pure crystalline OSCs by considering the details of the electronic band dispersions beyond effective mass approximation, with the full consideration of energy, ***k***-vector, and band index dependence of the relaxation time. This leads to very different predictions in comparison to a relaxation time, which does not depend on the carrier energy^43^ but is supported by the agreement with the experimental vibrational spectra and the temperature dependence of the mobility, around room temperature and below.^16^ It is worth mentioning that, in the most recent TLT implementation, the relaxation time related to the electron phonon scattering is proposed to be evaluated in a way much similar to ours, see in particular appendix A.3 of ref 41.

## 3 Results and Discussion

In the current section, we present the results of the study regarding the HT, LT, and TF polymorphs. The discussion starts with the results linked to the relaxed structures. The vibrational properties and Raman characteristics are then discussed in Section 3.2, with a focus on the different vibrational footprints of the molecules. The computed electronic structure is then presented in Section 3.2. Following this, Sections 3.3 and 3.4 describe, respectively, the EPC and the mobility calculations for the three polymorphs. Finally, the mobility results are analyzed with a discussion of the very different conduction characteristics of the molecule related to the different crystal forms and vibrational characteristics of the analyzed polymorphs.

### 3.1 Inherent Structures

Starting from the structures in the CCDC database,^53^ from which we obtained HT,^54–56^ LT,^54,55^ and TF^57^ CIF files, we first relax the atomic positions. The results are listed in Table 1. At the experimental lattice parameters of the polymorphs, the energy ranking is primarily governed by the VdW terms, demonstrating the importance of the dispersion contributions in stabilizing the structures. Next, we relaxed the cell parameters, to determine the inherent structures of the three polymorphs. The inherent structure of a system in a given configuration corresponds to the structure at mechanical equilibrium, found at the local minimum of the many-body potential energy hypersurface.^58^ This minimum is reached by the steepest descent minimization starting from the initial configuration. The unique local minima obtained from a set of experimental structures define the “natural” or “inherent” polymorphs that the system can adopt. The approach effectively removes the noise due to thermal expansion and is a reliable method for discerning experimental structures which may appear very similar.^13,15^ The energy values reported in Table 2 for the three calculated local minima are genuinely distinct, confirming that the three experimental structures correspond to different polymorphs. Moreover, the energy ranking is not determined by a single contribution but both electronic and dispersive interactions are relevant. As expected, the HT polymorph is the highest in potential energy, being in an enantiotropic relationship with the LT form, with a transition temperature of 463 K.^55^ Interestingly, the fact that the TF polymorph corresponds to a local minimum identifies it as a genuine structure, the existence of which is not induced by the presence of a substrate, despite having never been observed in bulk. Spectroscopically very similar to HT,^17^ TF is closer in energy to the LT polymorph. The genuinely different character of the three polymorphs is also signaled by the different Raman spectra, which are reported in Figure S1 and further described in Section 3.2.

**Table 1:** Total (*E*_tot_), Electronic (*E*_electr_), and VdW (*E*_disp_) Energies, in eV, after Relaxation of the Atomic Position at the Fixed Cell

atomic relax.	*E*_tot_	*E*_electr_	*E*_disp_
HT	–505.95925	–499.89885	–6.06040
TF	–506.01846	–499.79581	–6.22265
LT	–506.04048	–499.70373	–6.33675

**Table 2:** Total (*E*_tot_), Electronic (*E*_electr_), and VdW (*E*_disp_) Energies, in eV, after Unit Cell Relaxation

u.c. relax.	*E*_tot_	*E*_electr_	*E*_disp_
HT	–506.05713	–499.47794	–6.57919
TF	–506.06523	–499.52383	–6.54140
LT	–506.06801	–499.46723	–6.60078

### 3.2 Vibrational Properties

The vibrational properties of the three polymorphs were computed to gain insights into their structural dynamics. We begin by observing that the spectral patterns are clearly distinct, confirming that each describes the dynamics of a different local minimum, see Figure S1.

The Raman spectra, computed at the experimental unit cells and adjusted for the laser frequency and temperature, are displayed in Figure 1. The spectra are drawn as Lorentzian bands with a FWHM of 1/3 of the mean distance between the frequencies, chosen to conform to the experimental features.^16^ The peak positions agree with the experiments within a few cm^–1^, thus confirming the validity of our description in terms of pseudopotentials and electronic structures.^18,20,59,60^ The computed relative intensities are brought to satisfactorily agree with the experiments once the laser excitation frequencies and measurement temperatures are accounted for. This is obtained with the formula $I=I_{0}\frac{\nu }{{\left(\nu -\nu_{0}\right)}^{4}}\left[1-\exp\left(-\frac{h\nu }{kT_{0}}\right)\right]$ , where *I*_0_, ν_0_, *T*_0_, and ν are the measured intensity, excitation frequency, temperature, and vibration frequency, respectively, *h* is the Planck constant and *k* is the Boltzmann constant. *I* is the adjusted experimental intensity.^61^ Calculated and experimental wavenumbers are reported in Tables 3 and 4. To account for the frequency shifts observed for the lattice phonons computed at the experimental HT unit cell, determined at 478 K, by Siegrist et al.,^55^ vibrational modes have also been calculated for the structure reported by Campbell.^56^ While an approximately 2% increase in the cell volume in the high-temperature structure has no effect on the intramolecular modes, a softening of the order of a few wavenumbers characterizes the lattice phonons. A symmetry assignment was performed using the symmetry.py code, a postprocessing routine which employs the projector method.^62^ In the triclinic $P_{1̅}$ symmetry group, only modes of the A_g_ symmetry are Raman active. The low-frequency modes are almost completely intermolecular in character, and those of the A_g_ symmetry correspond to librations about the molecular inertia axis, while those of the A_u_ symmetry are translations.

**Figure 1:**
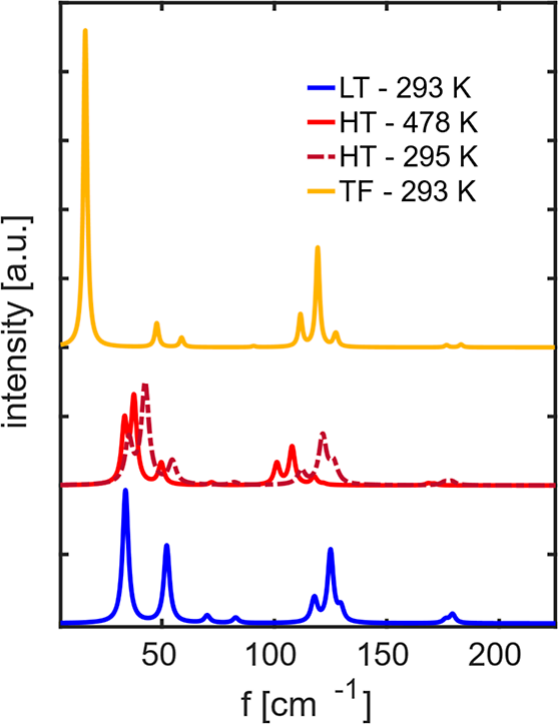
Calculated Raman spectra for the experimentally measured unit cells. The reported intensity is adjusted for the laser frequency and measurement temperature.

**Table 3:** Experimental Raman Frequency for HT,^63,64^ LT,^63,64^ and TF,^17,63^ Polymorphs Compared with the Computed Values; We Also Report the Polymorph Nomenclature Used in Other Works^17,63^ and the Name of the Main Author Whom the Polymorph Structure is Related to, and the Temperature at Which the Spectra or the Unit Cell (u.c.) Were Measured^b^

experimental polym. C (Campbell)				experimental polym. H(Holmes)				
80 K	300 K	computed HT (Siegrist) u.c. @ 478 K	computed HT (Campbell) u.c. @ 295 K	80 K	300 K	computed LT u.c. @ 293 K	experimental TF 300 K	computed TF u.c. @ 293 K
		25.6 (A_u_)	28.7 (A_u_)	44.9	33.1	33.9 (A_g_)		16.0 (A_g_)
49.4	36.4	33.5 (A_g_)	35.4 (A_g_)			36.8 (A_u_)		36.0 (A_u_)
54.9	45.5	37.7 (A_g_)	42.6 (A_g_)	65.5	52.2	52.2 (A_g_)	43.2	47.7 (A_g_)
		49.6 (A_u_)	52.9 (A_u_)			54.0 (A_u_)		48.0 (A_u_)
66.9	55.7	49.7 (A_g_)	54.6 (A_g_)			66.2 (A_u_)		80.9 (A_u_)
		58.3 (A_u_)	64.9 (A_u_)	84.3	69.8	70.2 (A_g_)	70^a^	90.8 (A_g_)
		72.1 (A_g_)	82.2 (A_g_)	99.1	88.1	82.8 (A_g_)		92.3 (A_u_)
		78.1 (A_u_)	87.6 (A_u_)			85.4 (A_u_)		111.6 (A_g_)
		82.2 (A_u_)	94.13 (A_u_)			95.6 (A_u_)		119.3 (A_g_)
99.8	94.4	101.1 (A_g_)	112.0 (A_g_)	132.2	122.5	117.1 (A_g_)		120.4 (A_u_)
126.7	115.1	107.9 (A_g_)	121.5 (A_g_)	136.2	133.2	118.0 (A_g_)		124.6 (A_u_)
		114.0 (A_u_)	122.1 (A_u_)			121.0 (A_u_)	122.3	126.8 (A_g_)
140.6	132.3	117.7 (A_g_)	126.2 (A_g_)			122.6 (A_u_)	132.3	127.5 (A_g_)
		119.2 (A_u_)	124.6 (A_u_)			124.7 (A_u_)		134.8 (A_u_)
147.5		121.9 (A_g_)	128.3 (A_g_)	144.1	149.5	124.9 (A_g_)		137.1 (A_u_)
		123.0 (A_u_)	127.1 (A_u_)					

a At *T* = 10 K.

b We show both gerade and ungerade modes for the sake of completeness. We restrict to the modes within the first bundle observable in the DOS. When computing the Raman frequency, we keep the experimental u.c. measured at the indicated temperature, thus a softening is expected for structures measured at high temperature.

**Table 4:**
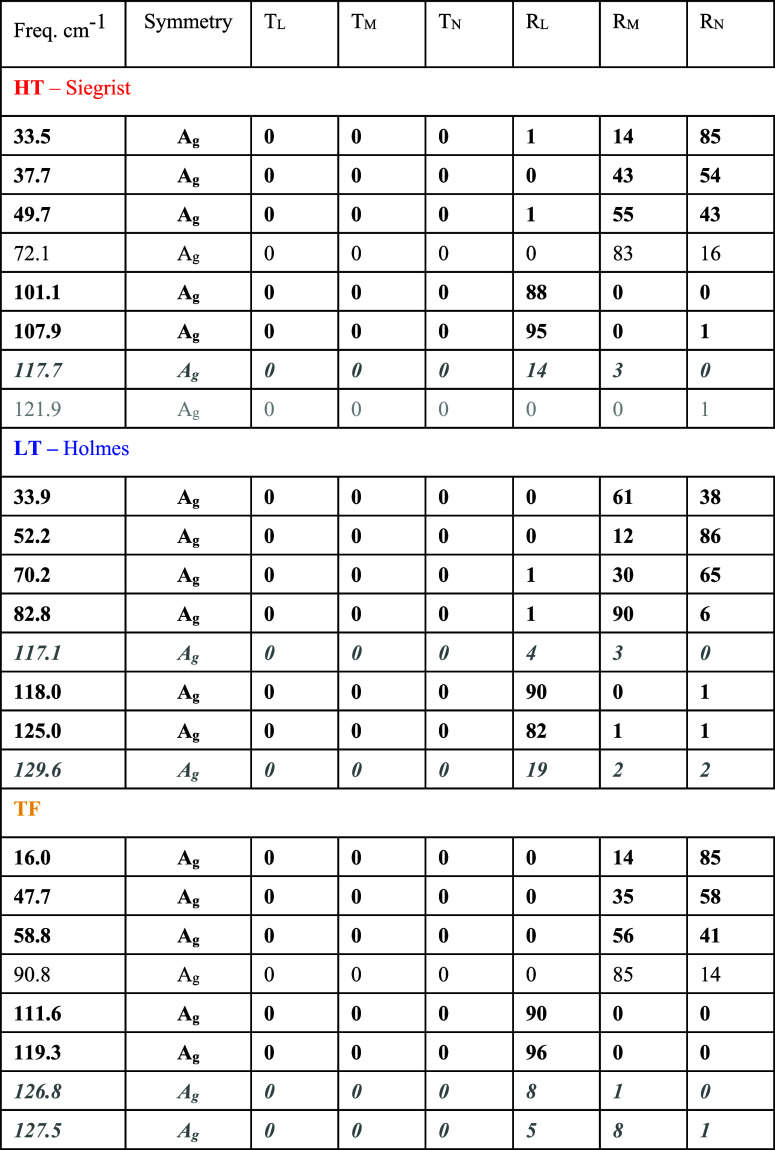
Calculated Wavenumbers of the Lowest Lattice Phonon Modes of HT, LT, and TF Polymorphs at Γ (with Symmetry) with Squared Translational (*T*) and Rotational (*R*) Components along and around the Three Main Inertia Axes (L, M, N), L and M in the Molecule Plane, and N Normal to the Molecular Plane^a^

a The modes in bold are the lattice phonons well visible in the Raman spectrum, the modes in bold and italic, dark grey, are mostly internal modes well observed in the Raman spectrum, the modes not in bold are lattice (black) or internal (faint gray) modes, which are poorly or not observed.

Considering the key role played by dynamics and disorder in shaping the charge transport characteristics of the pentacene polymorphs, and the relevance of Raman spectroscopy as a probe of the processes involved, it is worth analyzing in more detail the nature of the vibrational modes in the low-frequency region of the spectrum. Simulations confirm the characteristic patterns detected in the experiments, with HT and TF spectra more similar to each other than to LT.^12^ The eigenvector analysis reveals that the Raman modes below 120–130 cm^–1^ are predominantly of an intermolecular nature. As expected, the lowest energy vibrations involve mixed librations around the normal-to-plane (N) and short-in-plane (M) molecular inertia axes, which correspond to the highest moments of inertia. At higher energy, the modes correspond to almost pure rotations around the long-in-plane (L) molecular axis. The highest energy mode, however, exhibits a mixed intramolecular character, particularly in the HT and TF phases. It should be noticed that the calculations reproduce the experimental evidence that the high-frequency pattern is very similar for the three polymorphs, whereas major differences affect the lowest frequency range, where in fact, the TF Raman spectrum has never been clearly detected^17,63^ because in thin films HT and TF grow concomitantly and their appearance is found to be regulated by the film’s growth rate.^17^ As observed previously,^12^ such a feature must correspond to a characteristic of the pentacene polymorph structures and finds its explanation upon the analysis of the phonon dispersion curves.

Phonon dispersions and the corresponding DOS are reported in Figure 2. The dispersions along directions connecting high-symmetry ***q***-points are shown in Figure 2a–c. It is important to note that the first 20 modes are bundled together throughout the BZ. This feature is highlighted in Figure 2d, where the DOS of the polymorphs are reported. Thus, we consider them as 20 scattering channels, following the common approach in semiconductors,^30^ and, due to the dispersion through the BZ, we assign to each of them an effective phonon frequency ω_0,ν_.^16^ Because of their bundled character, we consider all of them and cut our region of interest at the first drop to zero of the phonon DOS.

**Figure 2:**
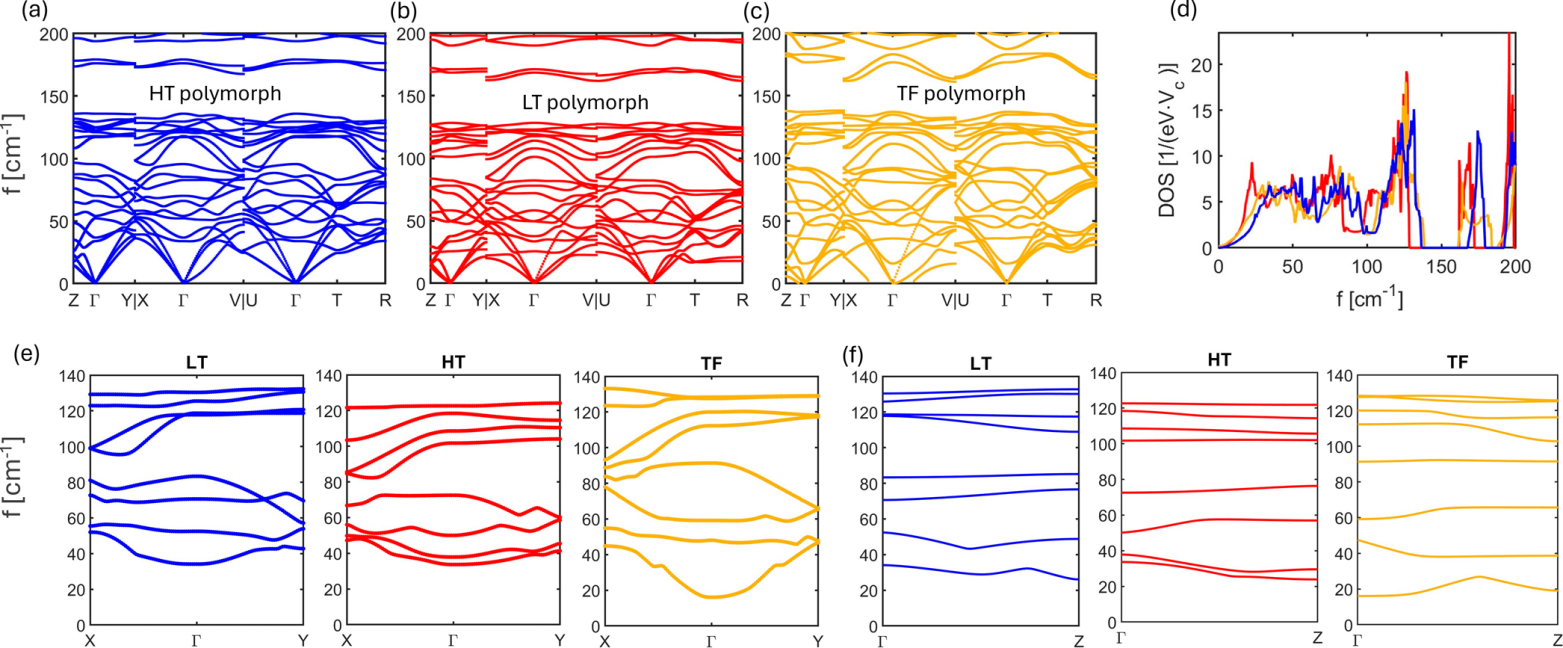
Phonon dispersions for the three polymorphs (a–c) and their DOS (d). (e,f) Dispersions of the Raman active modes of the three polymorphs, in the *ab* plane and along the *c* axis, respectively.

Vibrational spectroscopy provides lattice mode frequencies at the Γ point of the BZ, where all unit cells move in phase. The phonon dispersion curves, which describe the dependence of vibrational frequencies on the wavevector, offer insights into intermolecular interactions among different crystal cells. The analysis of the curves for the three pentacene polymorphs in Figure 2 reveals that all modes of intermolecular characters exhibit a significant dispersion along the a*b* directions (approximately in the *ab* crystal plane), demonstrating the strength of ab intralayer interactions. In contrast, only the lowest frequency modes, identified in the Raman spectrum as librations that tilt molecules, are dispersed along c*, the direction nearly perpendicular to the *ab* plane, where interlayer interactions are present. Librations about the *L* axis remain dispersionless in this direction, indicating their insensitivity to interlayer interactions, consistent with the fact that these phonons have the same description in the three polymorphs. This computational finding reinforces the results of lattice dynamics calculations based on empirical interatomic potentials for the TF structure and extends them to all pentacene polymorphs.

The occurrence of structural disorder in the c* direction disrupts the periodical interaction potential along that axis, thus preventing the establishment of a correlation length for collective phonon motions, that is, the distance over which the atomic displacements of the vibrations remain correlated. This mechanism has been invoked to explain both the vanishing intensities and the band broadenings affecting the lowest frequency lattice phonons observed in the films of pentacene polymorphs TF and HT,^17^ where the degree of the layer-by-layer order along the *c* axis depends on the deposition rate of the material. In contrast, the persistence of the high-frequency phonons indicates that the order on the *ab* plane, parallel to the film substrate, is preserved. In the actual samples, such a disorder is expected to influence the EPC along the c* direction in a number of ways. If the phonon mode cannot propagate coherently due to the structural disorder, then the electron interaction would be less effective. We further elaborate on this point in the EPC Section 3.4.

### 3.3 Electronic Structure

The electronic structure of pentacene has been investigated in detail elsewhere.^65^ We focus here primarily on a comparison of the electronic DOS of the three polymorphs, plotted in Figure 3. Solid lines are used for the topmost VB, while dash-dot lines denote the second one. The overlap region is shadowed, and the B_W_ of the topmost VB is reported in the legend. For the LT polymorph, the computed B_W_ compares well with the angle-resolved photoemission spectroscopy (ARPES) measurements of 206 meV (*T* = 110 K) and 175 meV (*T* = 75 K).^66,67^ ARPES measurements, presumably on LT phase samples, reveal bands narrower than what we obtain from DFT. However, we note that the larger bandwidth that we observe derives from the *X*–*Y* and *T*–*U* directions, which spans the (0,0,*k*_*z*_) planes with *k*_*z*_ = 0 and 0.5, respectively, according to the Bilbao Crystallographic Data Center nomenclature,^24,25^ thus following a line not passing from Γ. Since ARPES measurements are done along the reciprocal lattice direction from Γ to (11̅0), which corresponds to Γ–*V* in our plot, a smaller bandwidth detected by ARPES is expected.

**Figure 3:**
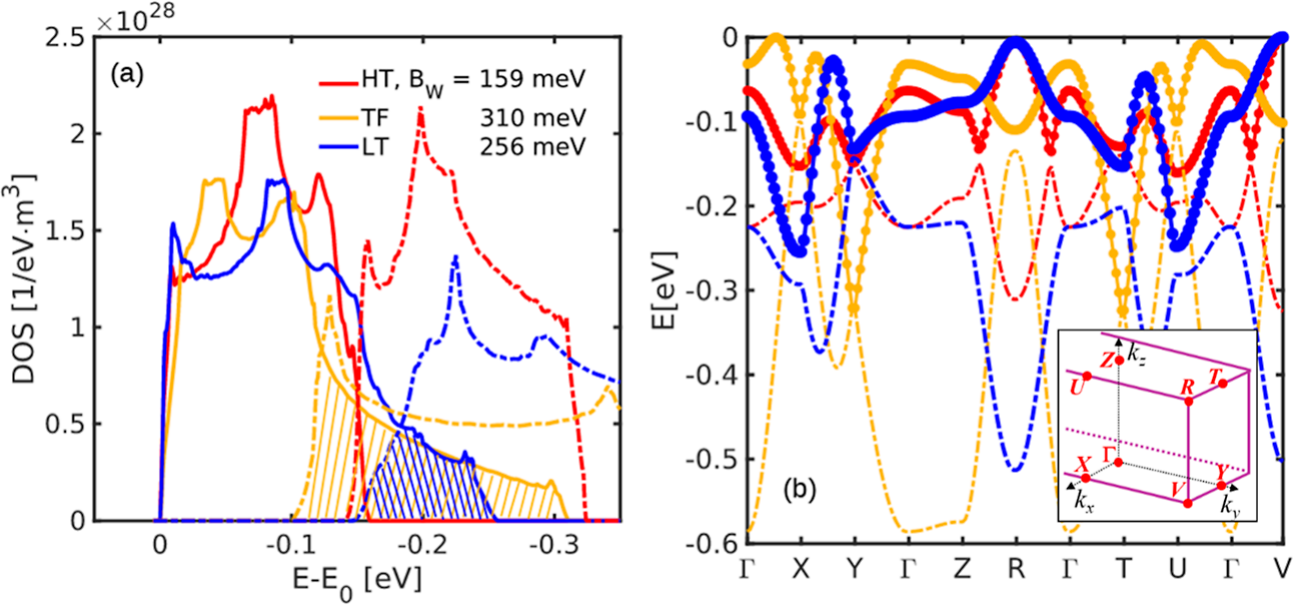
(a) VB electronic structure of the three pentacene polymorph, HT (red), TF (dark yellow), and LT (blue): comprehensive DOS with the indication of the bandwidth of the uppermost VB, and projections along certain directions; solid lines are for the uppermost band and dash-dot lines for the lower one. The inset in (b) reports the irreducible BZ wedge with the high symmetry points labeled as by the Bilbao Crystallographic Data Center.^24,25^

Furthermore, in the DOS plot in Figure 3, we highlight the overlap region between the two bands by shadowing it. This is the energy range where the interband scattering, eq 2, plays a role. We see that it is negligible for the HT phase because of the substantial absence of the overlap between the DOS, so that the fraction of carriers which can pass from one band to the other is negligible. The interband processes play a larger, yet minor, role in the LT because the DOS overlap involves a higher energy carrier. Differently, it is expected to be relevant in the TF polymorph, where it starts around 100 meV from the edge. Moreover, the EPC for the interband processes is seen as a vibrational modulation of the interaction between the two nonequivalent molecules inside the unit cell. Since B_W_ is comparable to the band separation, the interaction between the molecules inside the cell is similar to the one between molecules of other cells, i.e., we are not allowed to think in terms of the dimer. Thus, we expect the interband EPC is stronger also out of Γ, as we confirm in Figure S3.

### 3.4 EPC Parameters

In this section, we present the EPCs evaluated according to eqs 1–3. They have been computed with the Phonopy code on unit cells with atoms displaced along each eigenmode, following convergence tests that identified 0.0025 Å as an optimal displacement value, the results of the convergence tests are reported in Figure S2. In Table 5, we report the mode and the ***q***-point pair (***q***,ν) for which the EPC exceeds 85% of the highest value. We first observe that, at Γ, there are no modes that significantly contribute to the EPC. This demonstrates the importance of not confining the analysis to the BZ center,^68^ even though only Γ modes are spectroscopically accessible. Also, we notice that the HT polymorph has four modes with strong EPC, LT has only two modes, with EPC comparable to the HT, and TF has two modes with a stronger EPC than both of the other polymorphs. In Table 5, we also report the effective EPC for that mode and ***q***-point, evaluated as the EPC squared divided by the phonon energy, *EPC* ^2^/ℏω, after eq 10. We see that the effective scattering strength in the LT drops while in the other polymorphs stays high; as we see in Section 3.4, this influences the mobility.

**Table 5:**
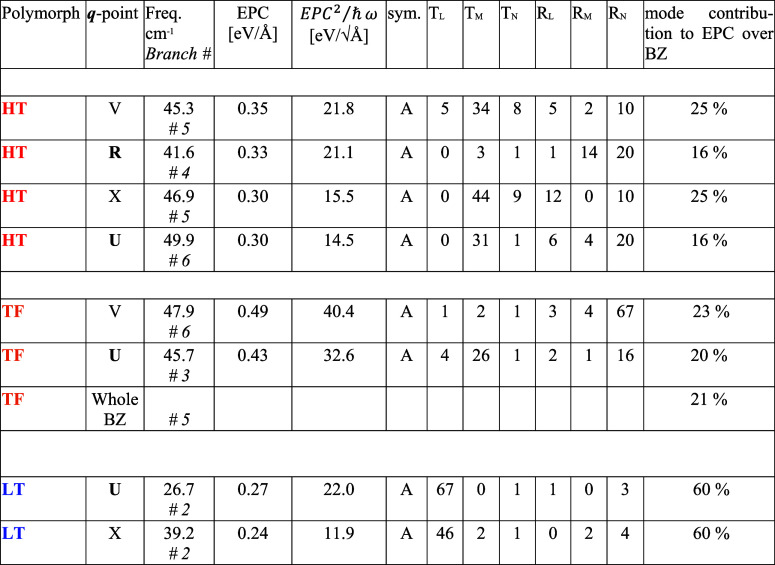
***q*** *-*points and Phonon Branch Numbers Featuring the Highest EPC Values in the Three Pentacene Polymorphs^a^

a The effective EPC as per eq 10 is reported as well, together with the mode symmetry and decomposition along the main molecular inertia axes. The contribution of the whole branch after the DOS weighted average over the BZ is also given. The ***q*** *-*points in bold have a component along the *z* direction.

We notice that in all three polymorphs, the largest EPC values are obtained for ***q*** *-*points which correspond to a direction with a *c* axis component, nominally U and R. The disorder effect described in the previous section causes a loss of coherence and disrupts the periodicity; the concept of BZ becomes meaningless along that direction. Hence, the EPC at R and U cannot be defined, with a decrease in the overall EPC, as per eq 3. However, vibrational degrees of freedom are expected to appear as a Gaussian distribution of frequencies without a dispersion relationship, i.e., lacking any definite ***q***-value dispersion relationship in the phase and frequency. These modes may contribute to EPC, but their effect cannot be precisely quantified at this level of description. If this approximate of “surrogate” EPC might be lower than the values calculated at the R and U points, which is likely, given that those represent some of the strongest couplings, then the overall EPC would be reduced, potentially leading to an enhancement in the carrier mobility. This can be the reason for the experimentally observed larger μ in films with a low interlayer correlation along c*, obtained at high growth rate.^75^ Such a situation can in fact be represented as a 2D arrangement of each single layer.

In addition to the (***q***,ν) pair with the highest EPC values, Table 5 reports the contribution to the effective EPC strength *EPC* ^2^/ℏω of the branch to which the pairs belong, following the calculation of the DOS-weighted average over the BZ as in eq 3. The (***q***,ν) pair EPC percentage strength is evaluated as the ratio between the *EPC* ^2^/ℏω of that mode at that ***q***-point and the total EPC computed as the sum of the *EPC* ^2^/ℏω for all of the (***q***,ν) pairs considered. It appears that, in HT and TF polymorphs, ∼60% of the EPC strength can be ascribed to three modes; for the TF phase, the mode #5 does not have particularly relevant individual (***q***,ν) pairs, but the overall branch accounts for a generally large EPC. Differently, in the LT polymorph, the 60% of the EPC strength is due to a single mode, with the largest contribution coming from zone boundaries.

The largest EPC occurs away from the Γ-point in all polymorphs, suggesting that spectroscopic methods relying solely on measurements at Γ ^6,69,70^ may provide an incomplete picture of the coupling landscape.

### 3.5 Mobility

In this section, we summarize the results achieved in terms of transport properties. First, we observe that the phonon-limited study reproduces the experimental rank. We use the approach in eqs 11–14 to obtain the mobility projected along arbitrary 3D orientations and compute an average mobility μ_r_ using more than 10^6^ random orientations. For the LT, HT, and TF, at room temperature, we find μ_r_ = 28.8, 6.5, and 5.7 cm^2^/(V s), respectively. To enforce our comparison with experimental results, in Table 6, we compare the computed mobilities with the experimental values of the best-case scenarios we found in the literature for a bulk single-crystal (s.c.),^71^ single-crystal field effect transistor (s.c.-FET),^72^ and thin-film field effect transistor (TF-FET).^73^ The calculated mobility is along the *b* axis, μ_*b*_, in the *ab* plane, μ_⟨*ab*⟩_, and also averaged over two million of random orientations, μ_r_.

**Table 6:** Comparison between Experimental and Calculated μ Values, the Crystal Direction Is at Subscript and Considered a Polymorph Phase Is in Parentheses^a^

system	experimental μ [cm^2^/(V s)]	calculated μ [cm^2^/(V s)]
s.c.	35 ^71^	μ_*b*_ = 52 (LT)
s.c.-FET	5.7 ^72^	μ_⟨*ab*⟩_ = 36 (LT) 5.9 (LT, *U*_b_ = 40 meV)
TF-FET	3 ^73^	μ_⟨*ab*⟩_ = 7.1 (TF)
		μ_r_ = 5.7 (TF)
		μ_⟨*ab*⟩*_ = 4.2 (TF/HT = 1:1, *U*_b_ = Δ*E*_v_^HT^ = 32.5 meV)
		μ_⟨*ab*⟩*_ = 1.6 (TF/HT = 1:1, *U*_b_ = 0.1 eV)

a When eq 16 is included, it is indicated. The asterisk indicates a mixture treated as from eq 15.

In Table 6, for the TF-FET case, we report also the case of a mixture of two polymorphs, where the mixture is treated as an inhomogeneous two-component medium,^74^ leading to

15 $$\mu =\mu_{1}^{f_{1}}\mu_{2}^{{1-f}_{1}}$$

where 1 and 2 are the two phases and *f*_1_ is the volume fraction of the first phase. In our estimates in Table 6, we assumed that TF and HT coexist^17^ in equal amounts (*f*_1_ = 0.5). Also, the different islands can have different structures at the coalescence boundary, with an impact on the resulting mobility.^75^ Thus, we phenomenologically introduce a scattering term with a characteristic energy equal to the energy distance between the top-valence band and other satellite valleys at a lower energy in the HT phase, Δ*E*_v_^HT^, which is 32.5 meV. We consider only HT, since TF appears not to have such satellite valleys, but only a valence band maximum without relative maxima. We treat such boundary scattering on the footing of the Fermi golden rule, using the characteristic energy as scattering potential *U*_b_ and the energy-dependent density of the states of the unit cell *g*_uc(*E*)_. Thus, we have a scattering time of τ_b(*E*)_ for this boundary scattering

16 $$\frac{1}{\tau_{b\left(E\right)}}=\frac{2\pi }{\hbar }{\left(U_{b}\right)}^{2}g_{uc\left(E\right)}$$

We used eq 16 also to account, in a macroscopic and phenomenological way, of structural defects in single crystals, where an effective barrier of ∼40 meV brings the phonon mobility in the measured range. Lastly, after the Kelvin probe microscopy (KPM) measurements on in operando pentacene FET, which revealed a ∼0.1 V voltage drop at any coalescence boundary between islands,^3^ we used eq 16 with *U*_b_ = 0.1 eV, together with a 1:1 polymorph mixture. The obtained μ is lower than the value measured in FET with the polymeric gate,^73^ but is closer to what is measured in the same experiment of KPM measurements,^3^ which featured oxide gate dielectrics. Thus, our preliminary results suggest that the strong impact of the dielectric/OSC interface^76^ occurs also at the level of crystal quality and defectivity of the OSC.

## 4 Conclusions

We employed a robust first-principles approach to investigate why closely related polymorphic systems, such as the three known crystal forms of pentacene, may, in fact, display markedly different charge mobilities. Our analysis of nonlocal electron–phonon couplings over the full BZ reveals that even structurally similar polymorphs can be characterized by distinct “killer” phonon modes^6^ Thus, we challenge the notion of killer phonon, i.e., the existence of a dominant vibrational mode. Instead, our studies reveal that the mobility is limited by more modes with diverse wavevectors, responsible for the scattering of the charge carriers and limiting the mobility. Importantly, these modes are not restricted to the BZ center detected by the spectroscopic methods (i.e., they possess nonzero wave-vectors). Even in the LT polymorph, where 60% of the effective EPC strength is ascribed to one phonon branch only, there are two dominant phonons belonging to the same branch but a different wave-vector. Since the identity and character of these detrimental phonons appear to depend even on structural details in closely related forms, their identification remains a complex task that requires a rigorous theoretical framework. What we can deduce at present is that, in the system with larger mobility, the stronger EPC is for modes involving molecule translations along the long inertia molecular axis. However, to what extent this conclusion is general and how to derive molecule design indications are still open tasks.

Our phonon-limited mobility calculations accurately reproduce the experimental trend in the three pentacene polymorphs, with average mobility values of 28.8, 6.5, and 5.7 cm^2^/(V s) for LT, HT, and TF phases, respectively. These values are consistent with the highest experimental mobility recorded for single crystals, FETs, and thin films. Importantly, we also modeled mixed-phase systems and incorporated additional scattering mechanisms to account for boundary effects and structural defects by means of a phenomenological treatment based on Fermi’s golden rule. These results highlight how subtle structural differences, phase mixing, and interface effects can dramatically influence charge mobility in real devices.

Our analysis shows that the disorder along the c* axis can effectively confine phonons in a quasi-2D system by suppressing modes with wave vectors *q* oriented out of the ab plane. This confinement reduces electron–phonon scattering processes, and thus the effective EPC, provided that the structural order is preserved in the ab plane and interface scattering is minimal. In the 2D thin-film polymorph, the stacking direction restricts out-of-plane vibrational motions, lowering the number and amplitude of low-frequency phonons that couple the most strongly to charge carriers. The resulting suppression of dynamic disorder stabilizes intermolecular transfer integrals, decreases carrier scattering events, and provides a physical explanation for the enhanced band-like mobility observed in the 2D phase compared to the 3D thin film. Analogous to the quantum confinement in inorganic semiconductors, this restricted phonon bandwidth highlights how structural dimensionality governs electron–phonon coupling and charge transport in organic semiconductors.

## Supplementary Material



## Data Availability

The data underlying this study are openly available in Zenodo with the following DOI: 10.5281/zenodo.17368135.

## Notes

Raman spectra of the fully relaxed structures; convergence test results for the EPC computation for all the HT, TF, and LT polymorphs; and calculated wavenumbers of the lowers lattice phonon modes of the HT, LT, and TF polymorphs at the center of the BZ with their symmetry (PDF)
