# Supplementary material for: Electrons and Phonons in Pentacene: Coupling Patterns Reveal the Microscopic Origin of the Phonon Limited Mobility
Source: J Phys Chem C Nanomater Interfaces. 2025 Nov 29;129(49):21738–50. doi: 10.1021/acs.jpcc.5c04906 (PMC12707224; doi:10.1021/acs.jpcc.5c04906)
Supplement: Supplementary file 1 [file jp5c04906_si_001.pdf]

Supplementary information

Electrons and phonons in pentacene: coupling patterns reveal the microscopic origin of the phonon limited mobility

Luca Gnoli[1,*], Elisabetta Venuti[2], Tommaso Salzillo[2], Matteo Masino[3], Patrizio Graziosi[1]

[1] ISMN – CNR, Consiglio Nazionale delle Ricerche, via Gobetti 101, 40129, Bologna, Italy

[2] Dipartimento di Chimica Industriale "Toso Montanari", Università di Bologna, via Gobetti 85, 410129, Italy

[3] Dipartimento di Scienze Chimiche, Della Vita e Della Sostenibilità Ambientale & INSTM-UdR Parma, Parco Area delle Scienze, 17/A, 43124 Parma, Italy

* lucagnoli@cnr.it

The present S.I. contains the Raman spectra of the inherent structures, the decompositions of both Raman- and IR-active modes on the molecular inertia axes, the convergence tests for the EPC, and the EPC values for the inter-band processes.

We present the Raman spectra of the fully relaxed structures, i.e. the inherent structures. The spectral patterns are clearly distinct, confirming that each describes the dynamics of a different local minimum. The inset in Fig. S1 highlights the frequency region of the lattice phonons, where the major differences arise due to the different intermolecular interactions that characterize each structure.

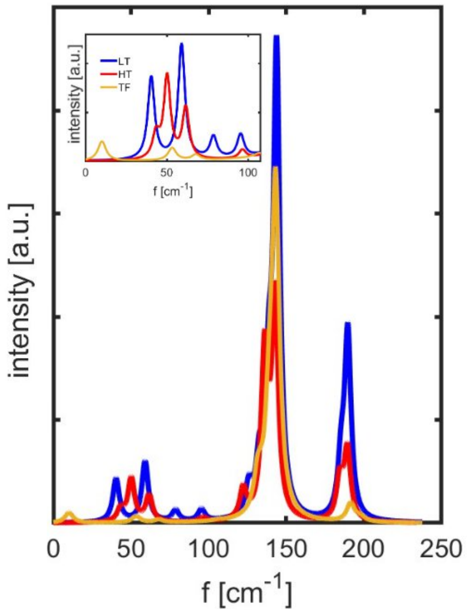

Figure S1: Raman spectra for the completely relaxed structures. The HT phase is the one measured at 478 K.

The convergence tests for the EPC* (sum of all the EPC values) in respect of the displacement along the vibrational coordinates in eq.s (1) and (2), are reported in Figure S2 for HT, TF, and LT polymorphs, respectively. Here, VB and CB indicates Valence and Conduction bands, the subscript numbers refer to the order of the band in respect of the band gap, i.e. the VB_1 has the maximum at the band edge, above VB_2 maximum, and CB_1 has the minimum at the band edge, below CB_2 minimum. The subscript 'inter' refer to the EPC for inter-band scattering.

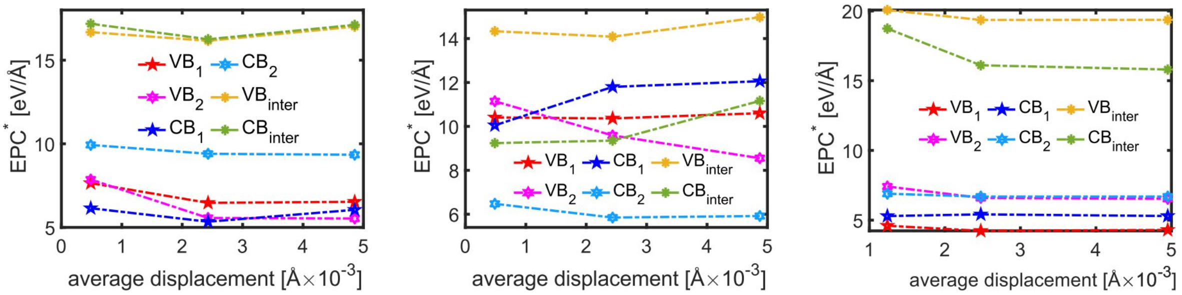

Figure S2: convergence of the EPC with the average atomic displacement, for HT, TF and LT polymorphs from left to right. The EPC is reported as sum of all the contributions, EPC*.

Table S1: calculated wavenumbers of the lowest lattice phonon modes of HT, LT and TF polymorphs at Γ (with symmetry) with squared translational (T) and rotational (R) components along and around the three main inertia axes (L, M, N), L and M in the molecule plane, N normal to the molecular plane.

Freq. cm^{-1}	Symmetry	T_L	T_M	T_N	R_L	R_M	R_N
**HT**							
25.6	A_u	94	1	0	0	0	0
33.5	A_g	0	0	0	1	14	85
37.7	A_g	0	0	0	0	43	54
49.5	A_u	3	1	0	0	0	0
49.7	A_g	0	0	0	1	55	43
58.3	A_u	2	77	19	0	0	0
72.1	A_g	0	0	0	0	83	16
78.1	A_u	1	5	19	0	0	0
82.2	A_u	0	15	60	0	0	0
101.1	A_g	0	0	0	88	0	0

107.9	A_g	0	0	0	95	0	1
117.7	A_g	0	0	0	14	3	0

LT

33.9	A_g	0	0	0	0	61	38
36.8	A_u	95	1	0	0	0	0
52.2	A_g	0	0	0	0	12	86
54.0	A_u	3	1	0	0	0	0
66.2	A_u	1	79	19	0	0	0
70.2	A_g	0	0	0	1	30	65
82.8	A_g	0	0	0	1	90	6
85.4	A_u	1	1	3	0	0	0
95.6	A_u	0	18	77	0	0	0
117.1	A_g	0	0	0	4	3	0
118.0	A_g	0	0	0	90	0	1
125.0	A_g	0	0	0	82	1	1
129.6	A_g	0	0	0	19	2	2

TF

16.0	A_g	0	0	0	0	14	85
36.0	A_u	94	1	1	0	0	0
47.7	A_g	0	0	0	0	35	58
48.0	A_u	3	1	0	0	0	0
58.8	A_g	0	0	0	0	56	41
66.2	A_u	1	76	20	0	0	0
80.9	A_u	0	5	16	0	0	0
90.8	A_g	0	0	0	0	85	14
92.3	A_u	2	17	62	0	0	0
111.6	A_g	0	0	0	90	0	0
119.3	A_g	0	0	0	96	0	0
126.8	A_g	0	0	0	8	1	0
127.5	A_g	0	0	0	5	8	1

In Figure S3 we plot the EPC strength for the inter-band processes as per eq. (2) in the manuscript, for the three polymorph following the color code used in the manuscript: blue for the LT, red for the HT, dark yellow for the TF. These EPC parameters are mode and ***q***-point dependent; in the x-axis we report the considered 20 modes, for each ***q***-point as indicated. We see that for LT and HT, some of the stronger EPC values are at Γ, whereas for the TF polymorph, where the inter-band processes are more important for the large bands overlap, the inter-band EPC is stronger at the zone boundaries.

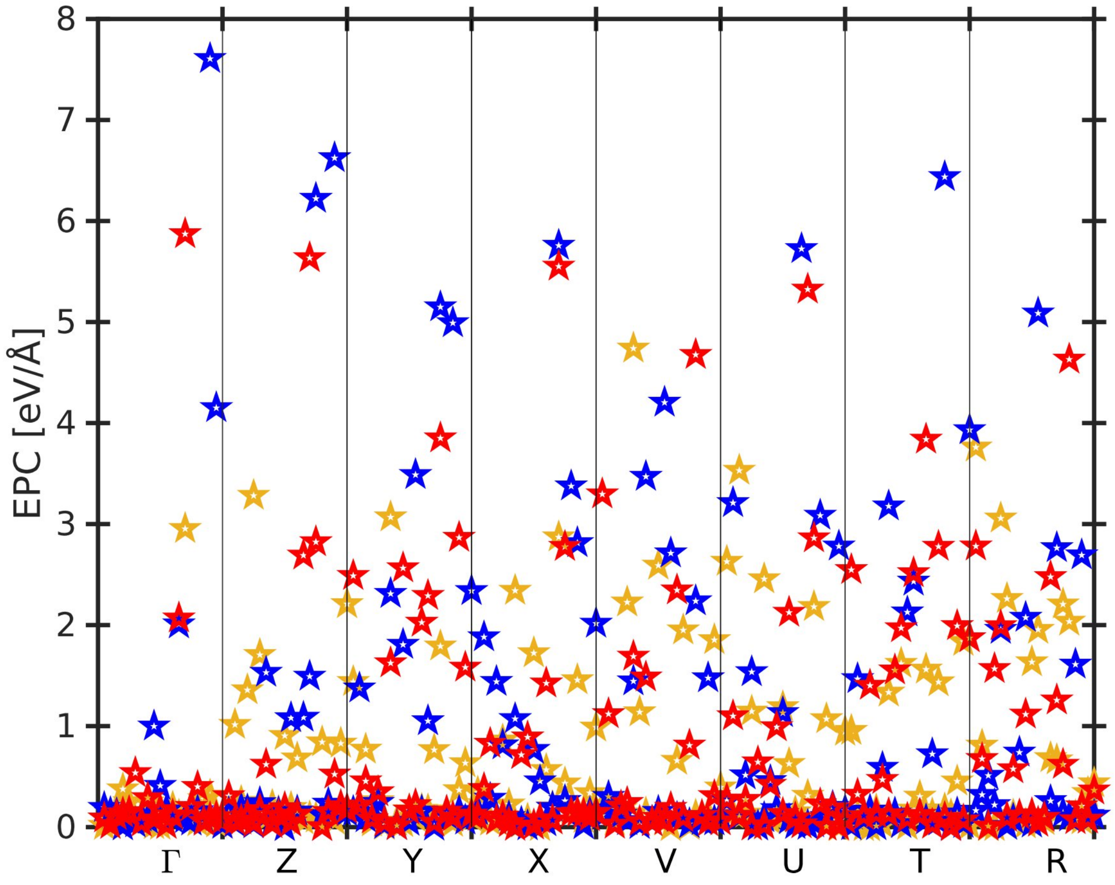

Figure S3: mode and ***q***-point dependent EPC parameter for the inter-band processes, eq. (2) in the manuscript. We report the values for the lowest 20 modes, which are the ones considered in the charge transport simulations.
